# Multi-Task Deep Learning Approach for Simultaneous Objective Response Prediction and Tumor Segmentation in HCC Patients with Transarterial Chemoembolization

**DOI:** 10.3390/jpm12020248

**Published:** 2022-02-09

**Authors:** Yuze Li, Ziming Xu, Chao An, Huijun Chen, Xiao Li

**Affiliations:** 1 Center for Biomedical Imaging Research, School of Medicine, Tsinghua University, Beijing 100084, China; liyz17@mails.tsinghua.edu.cn (Y.L.); xuzm20@mails.tsinghua.edu.cn (Z.X.); 2 Department of Minimal Invasive Intervention, Sun Yat-sen University Cancer Center, Guangzhou 510060, China; anchao@sysucc.org.cn; 3 Department of Interventional Therapy, Chinese Academy of Medical Sciences and Peking Union Medical College, Beijing 100021, China

**Keywords:** treatment outcome, liver neoplasms, deep learning

## Abstract

This study aimed to develop a deep learning-based model to simultaneously perform the objective response (OR) and tumor segmentation for hepatocellular carcinoma (HCC) patients who underwent transarterial chemoembolization (TACE) treatment. A total of 248 patients from two hospitals were retrospectively included and divided into the training, internal validation, and external testing cohort. A network consisting of an encoder pathway, a prediction pathway, and a segmentation pathway was developed, and named multi-DL (multi-task deep learning), using contrast-enhanced CT images as input. We compared multi-DL with other deep learning-based OR prediction and tumor segmentation methods to explore the incremental value of introducing the interconnected task into a unified network. Additionally, the clinical model was developed using multivariate logistic regression to predict OR. Results showed that multi-DL could achieve the highest AUC of 0.871 in OR prediction and the highest dice coefficient of 73.6% in tumor segmentation. Furthermore, multi-DL can successfully perform the risk stratification that the low-risk and high-risk patients showed a significant difference in survival (*p* = 0.006). In conclusion, the proposed method may provide a useful tool for therapeutic regime selection in clinical practice.

## 1. Introduction

Hepatocellular carcinoma (HCC) is one of the leading causes of cancer mortality worldwide, with more than 800,000 deaths reported annually [1,2,3,4]. HCC patients at an early stage are encouraged to perform curative therapies, such as liver resection, transplantation, and local ablation, observing the prolongation of overall survival (OS) [5,6]. However, more than half of patients with HCC are already in the intermediate and advanced stage for the initial diagnosis, and palliative treatment is the first choice [7]. According to the Barcelona Clinical Liver Cancer (BCLC) staging system, transarterial chemoembolization (TACE) is used as first-line therapy for patients at stage B. Patients with an objective response (OR) after the first session of TACE can obtain the survival benefit [8]. However, some patients still suffer a poor prognosis due to the complex heterogeneity of the tumor microenvironment [9,10]. Therefore, developing a prediction model for OR and OS of HCC patients who underwent TACE may have huge clinically significant for managing patients in this precision medicine era.

At present, several scoring systems have been proposed to predict the outcome of TACE for HCC patients. For example, Sieghart et al. developed the Assessment for Retreatment with transarterial chemoembolization (ART) score [11], which integrated radiologic tumor response, Child–Pugh increase, and aspartate aminotransferase increase to perform the staging. Hucke et al. proposed the selection for TACE treatment (STATE) score [12], measuring the serum-albumin level, tumor load, and C-reactive protein level to identify patients who were suitable or unsuitable for the first TACE. Furthermore, Granito et al. recently demonstrated that the post-TACE increase in transaminases could represent an independent factor for a complete response to TACE in patients with early and intermediate stage HCC [13]. This study may suggest a simple clinical tool associated with TACE’s efficacy to improve management and treatment planning. However, these scores are not widely used in clinical and are limited by the unsatisfied predictive accuracy. Moreover, researchers introduced quantitative imaging-based methods, such as radiomics approaches, to predict the OR of the TACE using high-throughput features extracted from computed tomography (CT) or magnetic resonance imaging (MRI) data [14,15,16]. Though these methods achieved considerable predictive ability, they rely on hand-crafted feature extractors and manual tumor segmentation, where the performance and efficiency can be further improved.

Machine learning, as a big data-driven approach, is widely used in the medical field [17], including liver tumor segmentation [18,19,20,21,22,23] and outcome prediction after TACE treatment [24,25,26,27,28,29]. For the former, researchers developed multi-layer convolutional networks, fully convolutional networks, and encoder–decoder structures, with which more and more information contained in images was utilized. For the latter, deeper and deeper neural networks were applied to extract features from the tumor region, aiming to provide more accurate outcome prediction. However, existing methods only utilized the deep neural network to perform a single task, ignoring the combination of these two interconnected tasks: tumor segmentation and outcome prediction. Here, we aimed to develop a tumor-aware deep neural network for multi-task learning towards both the TACE outcome prediction and tumor segmentation. We hypothesized that combining these two interconnected tasks in a unified model could behave better than only doing any single one of them. The network was constructed on a large cohort of HCC patients with TACE treatment and backed by external testing to demonstrate the effeteness and robustness of the proposed method.

## 2. Materials and Methods

### 2.1. Study Population

This study retrospectively enrolled patients with HCC who underwent TACE between May 2014 and December 2019 at two hospitals. The whole protocol was approved by the institutional ethics board, and the written informed consent was waived because of the retrospective nature of this study. All procedures involving human participants were performed following the 1975 Helsinki declaration and its later amendments.

In this study, HCC was confirmed by the European Association for the Study of the Liver (EASL) or the American Association for the Study of Liver Disease (AASLD). Specifically, the presence of arterial enhancement on contrast-enhanced CT (CECT) or contrast-enhanced MRI (CEMRI) of a nodule 2 cm or larger with subsequent washout on the portal or delayed phases was considered the HCC. CEMRI was recommended due to its high sensitivity [30]. Biopsy was performed if the nodule did not show typical features in images.

**Figure 1:**
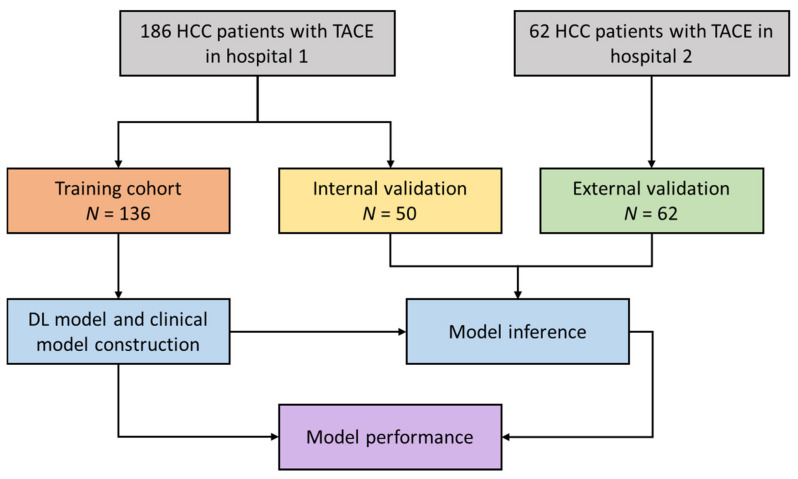
The diagram of the patient inclusion, model construction, and performance evaluation.

A total of 248 patients have analyzed in this study according to the following inclusion criteria: (1) age of the patient was equal to or older than 18; (2) BCLC stage A or B; (3) the CECT was performed within one month before the first session of the TACE; and (4) follow-up CECT or CEMRI was obtained two months after the treatment to determine the tumor response to TACE. The exclusion criteria were as follows: (1) other treatments such as resection, ablation, or transplantation were conducted before TACE; (2) presence of macrovascular invasion or extrahepatic metastasis; and (3) Child–Pugh C. Patients in hospital 1 were randomly divided into training and internal validation cohorts, and patients in hospital 2 were used as the external testing cohort (Figure 1).

Clinical variables for each patient were collected from the medical records, including 4 groups of data: demographics and clinical characteristics variables (sex, age, hepatitis B); laboratory findings (alpha-fetoprotein (AFP), alanine aminotransferase (ALT), aspartate aminotransferase (AST), albumin (ALB), prothrombin time (PT), total bilirubin (TBil), platelet (PLT), Child–Pugh class and BCLC grade); tumor characteristics (tumor number and tumor size); and treatment and follow-up information.

### 2.2. TACE Procedure and Follow Up

TACE treatment was decided by two experienced interventional radiologists with more than 10 years of TACE experience and approved by the patients. The TACE procedure was guided using digital subtraction angiography (Philips, type FD 20 1250 mA, Amsterdam, Netherlands). A 5-Fr micro-catheter (Terumo, Tokyo, Japan) was used to assess the feeding artery. Superselective embolization of the artery directly supplying the tumor was carried out with a microcatheter whenever necessary. Emulsion, which consisted of 10–20 mL lipiodol, 30–50 mg lobaplatin, and 20–40 mg epirubicin was injected slowly until the offending vessel occluded [31,32].

To determine the subsequent treatment, the CECT or CEMRI were conducted 4–8 weeks after TACE to evaluate the effectiveness and the tumor status. TACE can be discontinued when the residual tumor or new lesions are not found. In comparison, the patient can choose the “on-demand” TACE procedure with the presence of the vital tumor or recurrence.

The OR of TACE was determined by two interventional radiologists with more than 6 years of TACE operation experience according to the post-operative CECT with modified Response Evaluation Criteria in Solid Tumors (mRECIST) criteria, as recommended in [33]. Four categories of outcome were defined, including complete response (CR), partial response (PR), stable disease (SD), and progression disease (PD). CR and PR can be further classified into objective response (OR) group, while SD and PD were classified into non-response (Non-OR) group [34,35]. OS was defined as the period between the initial TACE treatment and all-cause death.

### 2.3. Image Acquisition and Pre-Processing

The detailed CECT imaging protocol can be found in Supplementary Materials Note S1. The arterial phase (AP) and portal venous phase (PP) of CECT images were used in this study. The normalization of the image was performed using the nearest interpolation method [36] to obtain 1 × 1 × 1 mm^3^ spatial resolution and using the Z-score method on the image intensity to 0–1 value. Then, the image was processed by the nnUNet [37] to obtain the initial tumor segmentation. One primary radiologist with 6 years of experience in liver imaging corrected the segmentation faults, while a secondary radiologist with 10 years of experience in liver imaging reviewed and adjusted the delineation. All adjustment was performed on in-house software coded by Python.

### 2.4. Deep-Learning Model Construction

**Figure 2:**
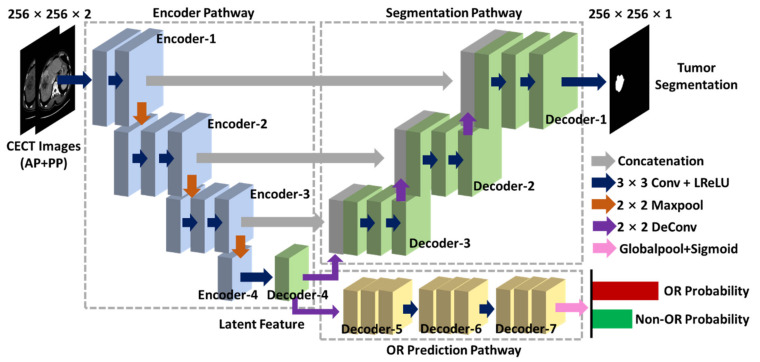
The structure of the proposed multi-DL (multi-task deep learning) model.

Here, a tumor-aware deep neural network for multi-task learning was developed to perform the OR prediction and tumor segmentation. The network was named multi-DL (multi-task deep learning) and its structure is shown in Figure 2 and Table 1. Inspired by the previous studies [38,39], we constructed the multi-DL model based on the encoder–decoder network where the encoder part (encoder pathway in the multi-DL) extracted the multi-scale information from the inputted images by down-sampling the resolution with pooling layer. In contrast, the decoder part (segmentation pathway) restored the resolution of feature maps layer by layer and integrated the multi-scale information through the skip connection. This encoder–decoder architecture was widely applied in liver tumor segmentation and showed promising results [18,19,20,21,22]. However, to the best of our knowledge, there is no relevant work to segment the liver tumor and predict the OR after TACE treatment simultaneously, which leaves a technique gap to fill. Therefore, we added the prediction pathway after the encoder pathway in multi-DL to realize the OR prediction in this study. There were two advantages of combining tumor segmentation and OR prediction into a unified network: first, existing OR prediction methods had to delineate the tumor region manually and then ran the algorithm on the image patch, which was time-consuming, while our multi-DL model can automatically locate the tumor area and generate OR prediction at the same time; and second, optimizing the tumor segmentation and OR prediction in the same network can obtain better performance than doing the single task, because these two tasks were interconnected and shared common characteristics which can be learned by the deep neural network. Additionally, the network output of the prediction pathway was a 0–1 value, indicating the probability of OR. The risk score was calculated by 1-OR probability and then used in the survival analysis to perform the risk stratification.

To verify the effectiveness of the multi-DL model, we compared the proposed method with other deep learning methods. For OR prediction, we compared ResNet50 applied in the study [26] and the single-DL-Pre model constructed of encoder pathway and prediction pathway. For tumor segmentation, we used the CNN model applied in the study [23] and encoder–decoder network in the study [18] as the comparison methods.

The loss function of the network was the combination of the cross-entropy loss and the dice loss [40]. The former was used for OR prediction, and the latter was for tumor segmentation. Dice similarity coefficient can be defined as follows:

Dice (pre, gt) = 2 × (pre ⋂ gt)/(pre + gt)

where pre denotes the predicted tumor region, gt denotes the ground truth tumor region and ⋂ denotes the intersection operation.

The model was trained for 200 epochs (the number of passes of the entire training dataset the deep-learning algorithm has completed) using the Adam optimizer (a widely used algorithm that modifies the attributes of the neural network, such as weights and learning rate) [41] with the learning rate of 1 × 10^3^. The implementation of the network was using the PyTorch framework (version 1.3.0) and Python (version 3.6) on a server equipped with a 6-core Intel CPU I7-6850K, a GPU TitanXp, and 32 GB memory.

### 2.5. Clinical Model Construction

The univariate analysis using logistic regression was firstly applied on all clinical variables, and those with significant differences between OR and non-OR (*p* < 0.05) were selected. Then, these variables were included in the multivariate logistic regression analysis to identify the independent risk factors associated with objective response (*p* < 0.05). Odds ratio and 95% confidence interval (CI) were calculated for each risk factor. The clinical model was constructed using the above independent risk factors using multivariate logistic regression algorithm [42].

### 2.6. Efficiency of Automatic Tumor Segmentation

We evaluated the efficiency of introducing automatic tumor segmentation into the model. Twenty patients were randomly selected from the training cohort and delineated by the radiologist, network processing, and network processing plus the manual adjustment. The averaged processing time was recorded and compared.

### 2.7. Statistical Analysis

The clinical variable distribution of the patients in the training, internal validation, and external validation cohorts were compared using Student’s *t*-test or chi-squared test. For the OR prediction, performance was evaluated using the receiver operating characteristic curve (ROC) analysis and the area under the curve (AUC). Quantitative indices including accuracy (ACC), sensitivity (SEN), specificity (SPE), positive predictive value (PPV), and negative predictive value (NPV) were also computed with the confusion matrix. Youden’s J statistic [43] was applied to determine the optimal operating points in ROC analysis.

The DeLong test was used to compare AUCs between different models. Survival curves were generated using the Kaplan–Meier method, and OS was compared between low- and high-risk patients with the log-rank test. For the tumor segmentation, the Dice coefficient and tumor segmentation time were compared using paired Student’s *t*-test. All statistical analyses were performed using R (version 4.0.4, Foundation for Statistical Computing, Vienna, Austria). A two-tailed *p*-value of less than 0.05 was considered as statistical significance.

## 3. Results

Table 2 shows the demographic of HCC patients in training (*n* = 136), internal validation (*n* = 50), and external testing cohorts (*n* = 62) with the mean age of 56.9, 57.9, and 57.7, respectively. 166 patients (66.9%) were in the BCLC stage B, and most of the patients (*n* = 217, 87.5%) were infected with hepatitis B. According to the mRECIST criteria, patients in OR and Non-OR groups were 82 and 166, respectively. The median follow up was 22.3 months (IQR: 10.9–28.5 months). There was no difference in distribution among training, internal validation, and external testing cohorts among all variables. For clinical variables, BCLC stage, tumor number, and tumor size were significantly associated with the OR status in the univariate analysis. Then, these three clinical factors were processed by the multivariate analysis to build the clinical model using the logistic regression model. Results showed that the BCLC stage, tumor number, and tumor size were independent risk factors (Table 3).

**Figure 3:**
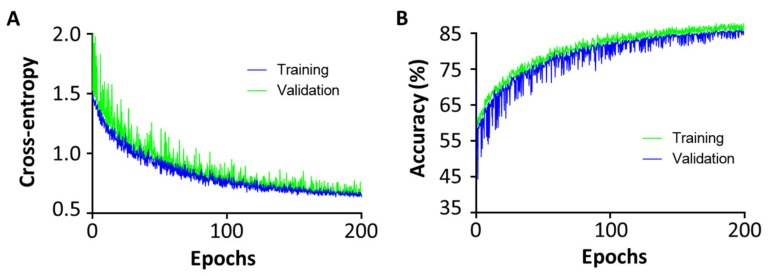
Training curve of multi-DL. (**A**) Cross-entropy vs. training epochs. (**B**) Accuracy vs. training epochs.

**Figure 4:**
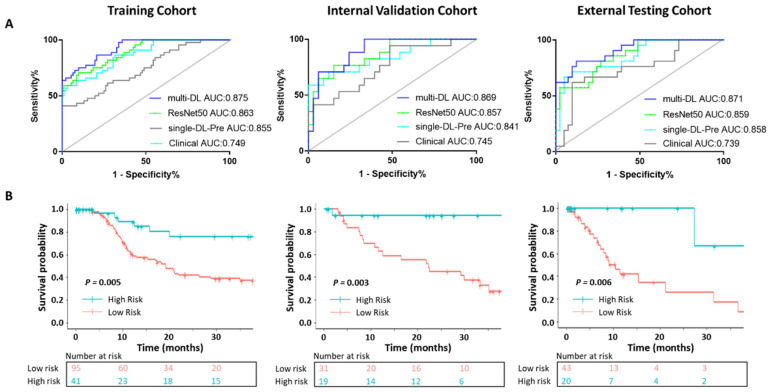
ROCs of OR prediction and risk stratification. (**A**) ROCs and AUCs of multi-DL, single-DL-Pre, ResNet50 and clinical model. (**B**) Survival curve of high- and low-risk patients stratified by the multi-DL model.

**Figure 5:**
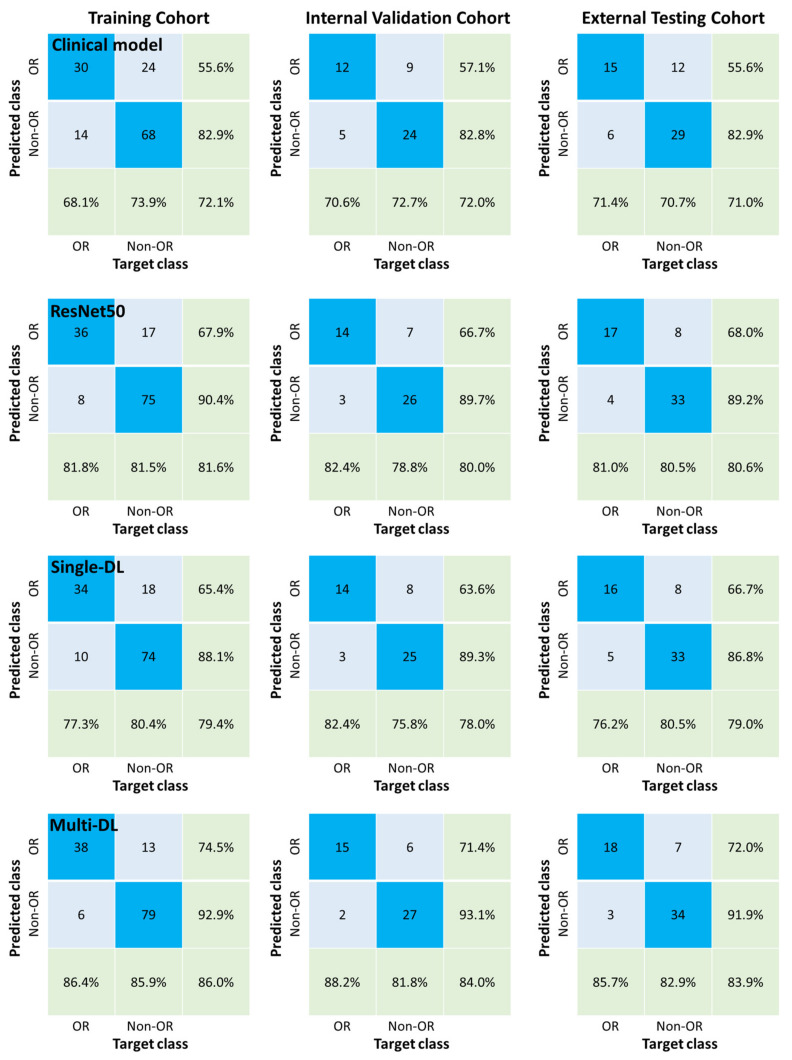
The confusion matrix for the clinical model, ResNet50, single-DL and multi-DL models.

Figure 3 shows the cross-entropy loss was close to 0.5 and accuracy was close to 0.85 after training of 200 epochs. Performances of different models in differentiation OR and Non-OR are shown in Figure 4A and Table 4. In the external testing cohort, the AUC of the multi-DL model was higher than both single-DL-Pre and ResNet50 which only performed the OR prediction (0.871 vs. 0.858 for single-DL-Pre, *p* = 0.073 and 0.871 vs. 0.859 for ResNet50, *p* = 0.065). Additionally, AUC of multi-DL was higher than the clinical model (0.871 vs. 0.739) with a significant difference (*p* < 0.01). Figure 5 shows the confusion matrices and quantitative indices that multi-DL obtained the highest ACC of 0.839, SEN of 0.857, SPE of 0.829, PPV of 0.720 and NPV of 0.919 among single-DL-Pre (ACC of 0.790, SEN of 0.762, SPE of 0.805, PPV of 0.667 and NPV of 0.868), ResNet50 (ACC of 0.806, SEN of 0.810, SPE of 0.805, PPV of 0.680 and NPV of 0.892), and the clinical model (ACC of 0.710, SEN of 0.714, SPE of 0.707, PPV of 0.556 and NPV of 0.829) in the external testing cohort.

The predicted OR and Non-OR patients were classified into low- and high-risk groups with the risk score threshold of 0.5. The survival curves are shown in Figure 4B. The proposed multi-DL can successfully perform the risk stratification where the low-risk and high-risk patients showed significantly different survival probability not only in training (*p* = 0.005) but also in the internal validation (*p* = 0.003) and the external testing cohort (*p* = 0.006).

**Figure 6:**
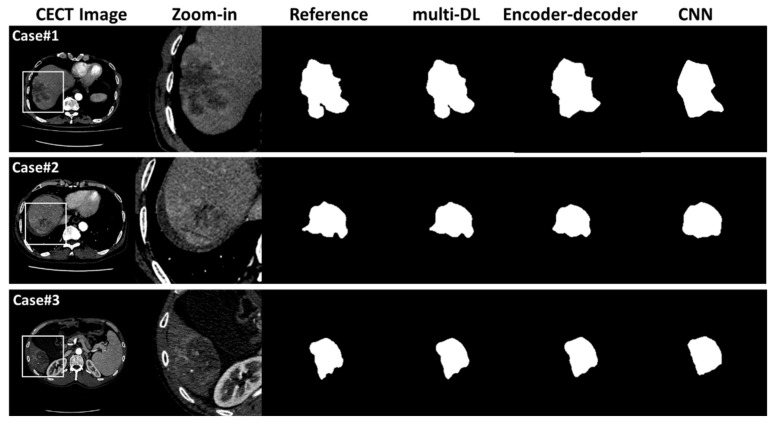
Tumor segmentation for three cases with reference manual segmentation, results of multi-DL, CNN, and encoder–decoder methods.

Reference tumor segmentation results are shown in Figure 6. Visually, the multi-DL method can generate a more accurate lesion boundary than CNN and encoder–decoder methods. For example, in case#1, multi-DL can successfully segment the tiny hollow of the tumor while other methods failed to restore this detail. In the quantitative analysis (Table 4), the dice coefficient of the multi-DL method was significantly higher than that of encoder–decoder (73.6% vs. 66.7%, *p* = 0.001) and CNN (73.6% vs. 63.2%, *p* < 0.001), which was in accordance with the observation result. For the tumor delineation time comparison, the manual segmentation was much slower than the deep neural network processing (1.0 s/slice vs. 30.5 s/slice, *p* < 0.001) and network processing plus manual adjustment (1.0 s/slice vs. 5.2 s/slice, *p* < 0.001), which demonstrated the high efficiency of the proposed method.

## 4. Discussion

In this study, a deep learning-based approach was proposed to simultaneously perform the accurate OR prediction in HCC patients with TACE treatment and automatically segment the tumor. The high performance of the proposed multi-DL method demonstrated combing two interconnected tasks (OR prediction and tumor segmentation) in a unified model could behave better than the single task.

TACE was a recommended initial treatment for HCC patients who could not receive the resection or ablation [44]. However, if patients were not appropriately selected, they could not benefit from the TACE procession, and the OS was not conferred. A previous study [45] reported that more than half of the patients had no OR to TACE. This ratio was 66.9% in our study, demonstrating the vital importance of the pre-operative prediction of OR. However, a recent study [46] reported that a restricted mean duration of response (DOR) might be a better end-point for decision-making. Still, the study only focused on two real trials with randomized phase 2 screening design, which needed further validations. Therefore, we chose OR as the end point to perform the prediction, considering the value of OR has already been demonstrated for clinical decision-making in routine practice and clinical trials [47].

Researchers have conducted relevant studies which adopted machine learning to predict OR after TACE. Abajian et al. used a supervised machine-learning method to predict the response to TACE and achieved an accuracy of 78%. Mähringer-Kunz et al. applied the CNN model to perform the survival prediction after TACE, and an accuracy of 77% was obtained. We extended the existing OR prediction framework based on previous studies by adding the tumor segmentation to form a unified model. In the proposed network, the encoder extracted and fused the inputted information to generate the latent feature, which was shared by the prediction and segmentation pathway. Therefore, the network may focus more on the tumor region and extract more cancer-related information when the network performed the OR prediction. Thus, multi-DL obtained higher accuracy in OR prediction (0.839) than ResNet50 (0.806) and the clinical model (0.710). Peng et al. recently used ResNet50 to predict OR after TACE on a large dataset including 789 patients and obtained a higher AUC of 0.97, which was higher than ResNet50 in our study (0.86). We inferred there were two reasons. Firstly, 562 patients’ data were used for training in Peng’s work, while only 136 patients’ data were used in our study. Large-scale training data can help the neural network learn more different features, so better results were obtained. Secondly, all CT images were reconstructed using a medium sharp reconstruction algorithm with a thickness of 1 mm in Peng’s study. However, in our research, CT images were collected from picture archiving and communication system (PACS) with DICOM format. They were reconstructed using different algorithms provided by different CT vendors and had different resolutions and thicknesses. Though the image pre-processing was performed in our study, CT images also had variations. Therefore, the neural network in Peng’s work may learn a relatively simple task while our model had to face a more complex condition, in which the performance of the model was degraded.

The multi-task learning brought another advantage: the automatic segmentation of the tumor. The dice of tumor segmentation of the multi-DL model was higher than compared methods (73.6% vs. 63.2% for CNN and 66.7% for encoder–decoder model) and showed comparable results to those of other studies. Budak et al. adopted a cascaded convolutional encoder–decoder neural network for tumor segmentation, which achieved the dice coefficient of 64.3% [18]. Adding the attention mechanism to U-Net, AHCNet obtained the dice of 0.734 for liver tumor segmentation on CT images [48]. Chlebus et al. proposed a fully convolutional neural network with object-based postprocessing for tumor segmentation and had dice value of 0.72 [20]. In this study, the multi-DL model encoder was used to extract and fuse the inputted information to generate the latent feature, which was shared by the prediction and segmentation pathway. Then, when the segmentation pathway processed the latent feature, the OR-related tumor characteristics such as tumor size, shape, and location may also benefit tumor segmentation, thus leading to higher performance than other compared methods. However, some works were reporting higher dice values for tumor segmentation. For example, Duc et al. proposed a 3D full resolution U-Net model for liver tumor detection and segmentation, which achieved dice of 0.81 in the test set. Due to the limitation of computation resources and training data, we adopted a 2D network in our study. Therefore, the spatial context information cannot be utilized, leading to degraded performance. In the future, we will explore the 2.5D network to use the inter-slice information but with less memory assumption [49].

Another point worth discussing was the efficiency of the proposed model. Since one HCC patient may have dozens of slices containing the tumor, radiologists had to manually delineate many CT images to perform the disease evaluation or surgery planning. Therefore, 6× or 30× of time reduction (30 s to 5 s or 30 s to 1 s) may significantly improve the efficiency in practice. With this deep-learning algorithm or intelligent software, interventional radiotherapy procedures can be optimized to reduce the radiation exposure of patients and interventional radiologists [50]. Additionally, multi-DL had few network parameters (~1.7 M) compared with ResNet50 (~27 M). Training the neural model with many parameters was expensive, requiring more computation resources and training data. From this perspective, our method had better ease of use because it can be transferred to other medical centers where the researchers can build their own clinical solutions using data of a regular size.

There remained some limitations in this study. First, the nature of the retrospective study may bring bias into the model construction, and a large prospective study with a longer follow-up period should be conducted. Second, only 2D images were processed by the neural network, while the spatial context information was not well utilized in our study [51]. Last but not least, the doxorubicin-loaded drug-eluting beads TACE (DEB-TACE) was widely used, and it was more cost-effective than the traditional TACE treatment [52]. Though we have already applied DEB-TACE in these two medical centers, the number of patients under this treatment was relatively small. In the future, we will include more patients with DEB-TACE to validate further the robustness and effectiveness of our proposed multi-DL method.

In conclusion, we developed and validated a multi-task deep learning approach for OR prediction and tumor segmentation in HCC patients with TACE treatment, backed by internal and external testing from multiple-center datasets. The high performance of the proposed method demonstrated that combing these two interconnected tasks in a unified model could behave better than other compared methods. Furthermore, the proposed model can successfully stratify the survival risk of HCC patients and may provide a useful tool of therapeutic regime selection in clinical practice.

## Figures and Tables

**Table 1:** The detailed network structure of multi-DL (multi-task deep learning).

Block Name	Layer	Parameter
Encoder-1	2 × (Conv2D + BN + LReLU) + Max-pooling	Conv: 3 × 3 × 32 filter, stride 1, same padding; Pooling: stride 2
Encoder-2	2 × (Conv2D + BN + LReLU) + Max-pooling	Conv: 3 × 3 × 64 filter, stride 1, same padding; Pooling: stride 2
Encoder-3	2 × (Conv2D + BN + LReLU) + Max-pooling	Conv: 3 × 3 × 128 filter, stride 1, same padding; Pooling: stride 2
Encoder-4	2 × (Conv2D + BN + LReLU) + Max-pooling	Conv: 3 × 3 × 256 filter, stride 1, same padding; Pooling: stride 2
Encoder-5	Conv2D + BN + LReLU	Conv: 3 × 3 × 128 filter, stride 1, same padding;
Encoder-6	Conv2D + BN + LReLU	Conv: 3 × 3 × 64 filter, stride 1, same padding;
Encoder-7	Conv2D + BN + LReLU	Conv: 3 × 3 × 32 filter, stride 1, same padding;
Decoder-1	(Conv2D + BN + LReLU) + (DeConv2D + BN + LReLU)	Conv: 3 × 3 × 32 filter, stride 1, same padding; DeConv: 3 × 3 × 32 filter, stride 2, same padding
Decoder-2	(Conv2D + BN + LReLU) + (DeConv2D + BN + LReLU)	Conv: 3 × 3 × 64 filter, stride 1, same padding; DeConv: 3 × 3 × 64 filter, stride 2, same padding
Decoder-3	(Conv2D + BN + LReLU) + (DeConv2D + BN + LReLU)	Conv: 3 × 3 × 128 filter, stride 1, same padding; DeConv: 3 × 3 × 128 filter, stride 2, same padding
Decoder-4	(Conv2D + BN + LReLU) + (DeConv2D + BN + LReLU)	Conv: 3 × 3 × 256 filter, stride 1, same padding; DeConv: 3 × 3 × 256 filter, stride 2, same padding

**Table 2:** Patient characteristics in the training, internal validation, and external testing cohorts.

	Training (*n* = 136)	Internal Validation (*n* = 50)	External Testing (*n* = 62)	*p*
Mean age (years)	56.9 ± 11.9	57.9 ± 10.9	57.7 ± 13.3	0.831
F/M ratio	14:122	1:49	4:58	0.221
Child–Pugh class				0.397
A	116 (85.3)	41 (82.0)	48 (77.4)	
B	22 (14.7)	9 (18.0)	14 (22.6)	
BCLC stage				0.129
A	50 (36.8)	18 (36.0)	14 (22.6)	
B	86 (63.2)	32 (64.0)	48 (77.4)	
HBV				0.730
Presence	117 (86.0)	45 (90.0)	55 (88.7)	
Absence	19 (14.0)	5 (10.0)	7 (11.3)	
ALB (g/L)	38.8 ± 4.1	38.2 ± 5.1	38.1 ± 5.8	0.589
ALT (U/mL)	65.7 ± 58.5	77.1 ± 93.8	68.8 ± 76.6	0.630
AST (U/mL)	111.4 ± 235.4	110.8 ± 89.2	125.0 ± 112.7	0.882
PT, seconds	12.2 ± 1.7	12.0 ± 1.0	12.5 ± 1.5	0.188
PLT × 10^9^/L	195.1 ± 78.1	203.8 ± 90.3	205.5 ± 120.0	0.715
TBil (umol/L)	18.6 ± 24.3	16.9 ± 8.1	17.9 ± 13.5	0.869
AFP (ng/mL)				0.202
≤400	71 (52.2)	31 (62.0)	40 (64.5)	
>400	65 (47.8)	19 (38.0)	22 (35.5)	
Tumor maximum diameter (cm)	9.0 ± 3.6	9.3 ± 3.9	9.5 ± 4.4	0.652
Multiple tumors				0.098
Single	54 (39.7)	19 (40.0)	15 (24.2)	
Multiple	82 (60.3)	31 (60.0)	47 (75.8)	
Tumor response				0.773
OR	44 (32.4)	17 (34.0)	21 (33.9)	
Non-OR	92 (67.6)	33 (66.0)	41 (66.1)	

Abbreviations: HBV, hepatitis B virus; AFP, alpha fetoprotein; ALT, alanine aminotransferase; AST, aspartate aminotransferase; ALB, albumin; PT, pro-thrombin time; TBil, total bilirubin; PLT, platelet; OR, objection response.

**Table 3:** Uni- and multivariable regression analysis of predictors of OR in the training cohort.

Clinical Variables	β	Odds Ratio (95% CI)	*p* Value	β	Odds Ratio (95% CI)	*p* Value
Mean age (years)	−0.014	0.986 (0.950–1.024)	0.470			
Sex (Female/Male)	0.142	1.152 (0.306–4.342)	0.834			
Child–Pugh class	0.139	1.150 (0.424–3.120)	0.784			
BCLC Stage (B/A)	−1.697	0.183 (0.062–0.542)	0.002 *	−1.556	0.211 (0.079–0.562)	0.002 *
HBV (Presence/Absence)	−0.587	0.556 (0.172–1.792)	0.325			
ALB (g/L)	−0.033	0.967 (0.864–1.084)	0.567			
ALT (U/mL)	−0.004	0.996 (0.987–1.004)	0.303			
AST (U/mL)	0.000	1.000 (0.996–1.003)	0.759			
PT, seconds	0.188	1.206 (0.835–1.743)	0.318			
PLT × 10^9^/L	0.004	1.003 (0.997–1.008)	0.371			
TBil (umol/L)	0.011	1.011 (0.981–1.041)	0.470			
AFP (>400 ng/mL/≤400 ng/mL)	−0.361	0.697 (0.297–1.634)	0.406			
Tumor maximum diameter (>5 cm/≤5 cm)	−1.399	0.247 (0.073–0.835)	0.024 *	−1.654	0.191 (0.065–0.562)	0.003 *
Multiple tumor (Single/Multiple)	1.298	3.664 (1.222–10.983)	0.020 *	1.059	2.884 (1.071–7.764)	0.036 *

* indicated *p* < 0.05.

**Table 4:** Performance for multi-DL and compared methods.

Method	AUC	ACC (%)	Dice (%)
OR Prediction			
Clinical model	0.739	71.0	N/A
ResNet50 [26]	0.859	80.6	N/A
Single-DL-Pre	0.858	70.9	N/A
Tumor Segmentation			
CNN [23]	N/A	N/A	63.2
Encoder–decoder [18]	N/A	N/A	66.7
Ours			
Multi-DL	0.871	83.9	73.6

N/A: Not applicable.

## Data Availability

The datasets used during the current study are available from the corresponding author on reasonable request.

## Supplementary Materials

The following supporting information can be downloaded at: https://www.mdpi.com/article/10.3390/jpm12020248/s1, Note S1: CECT Imaging Protocols.
