# Supplementary material for: Multi-Task Deep Learning Approach for Simultaneous Objective Response Prediction and Tumor Segmentation in HCC Patients with Transarterial Chemoembolization
Source: J Pers Med. 2022 Feb 9;12(2):248. doi: 10.3390/jpm12020248 (PMC8875107; doi:10.3390/jpm12020248)
Supplement: Supplementary file 1 [file jpm-12-00248-s001.zip › jpm-1543817-supplementary.pdf]

Multi-Task Deep Learning Approach for Simultaneous Objective Response Prediction and Tumor Segmentation in HCC Patients with Transarterial Chemoembolization

Supplementary Materials

Note S1: CECT Imaging Protocols

The CECT imaging were acquired using Siemens SOMATOM Sensation 64 CT, Philips Marconi MX8000, GE Discovery 750 HD, Philips Brilliance iCT, Siemens SOMATOM Force CT scanners. The detailed parameters were as follows: tube voltage, 120 kV; tube current, 100-500 mAs; rotation time, 0.5, 0.6, 0.75 second; detector collimation, 64 × 0.625 or 128 × 0.6mm; field-of-view, 360×360mm; matrix, 512 × 512; reconstruction thickness, 5mm. The iodinated contrast agent (Ultravist; Bayer Schering Pharma, Berlin, Germany) was injected into HCC patients with a dose of 70-100 ml and a rate of 3.0–3.5 mL/s. CECT images were acquired with arterial, portal venous and delay phase at 20–30, 50–60 and 110-120 seconds delay, respectively.
